# In the quicksand: the suspended lives of non-returnable migrants in Italy

**DOI:** 10.3389/fsoc.2026.1897781

**Published:** 2026-08-17

**Authors:** Francesca Cimino, Beatrice Grasso, Fabio Perocco

**Affiliations:** Department of Philosophy and Cultural Heritage, Ca' Foscari University of Venice, Venice, Italy

**Keywords:** irregular migration, limbo, migration, non-returnable migrants, precarity

## Abstract

Although, as in the rest of Europe, there is no precise data on the number of non-returnable migrants, in Italy an estimated 80% of third-country nationals who have been served with an expulsion order have not been returned for policy reasons or due to *de jure* (legal) or *de facto* (practical) factors. The category of “non-returnability,” first introduced in Italy in 1998, has been subject to various amendments over the years and subsequently divided into distinct categories. These changes have occurred alongside constant modifications in the legal status of migrants, whose rights have been expanded and restricted in line with the changing political priorities of those in power. However, the rights of non-returnable migrants have never been clearly defined, leaving them in an uncertain and paradoxical situation, particularly when it comes to accessing services, health, education, work, and housing, stuck in a limbo between an irregular and a regular migration status. Based on the Horizon Europe Project “MORE – Motivations, experiences and consequences of returns and readmissions policy (n° 101094107),” this article presents the findings on the condition of non-returnable migrants in Italy through an analysis of the relevant policy and legal frameworks, as well as interviews with experts, state agents, and migrants. The study reveals that non-returnability does not equate to regularization, thus hampering these migrants' socio-economic insertion; furthermore, it shows that there is much administrative arbitrariness in relation to this category, meaning that different non-returnable migrants find themselves in different social conditions and enjoying different social rights as a result of various factors (for example, the constant variability of policy framework or the local context). Trapped in a legal limbo and with their lives in a social limbo seemingly “on hold”—in terms both of their migratory status and their social situation—these un(der)documented migrants find themselves one step up from “irregular” undocumented migrants and one step below “regular” documented migrants, navigating uncertainty, precarity, and liminality.

## 1 Introduction

The increasingly restrictive, selective, and punitive migration policies of European states and the European Union (EU) have led to a general worsening of the conditions of migration, both during the journey and on arrival (Düvell, 2002; Finotelli and Ponzo, 2023; Migreurop, 2007). On the one hand, borders are fortified and externalized, migration routes are militarized, pushbacks are increased and legal channels for entering and remaining in European countries are reduced, forcing migrants into a position of “illegality.” On the other hand, there is an increase in return policies and readmission agreements, as well as detention in removal and repatriation centers, and a rise in voluntary or forced individual and collective returns. These are the two arms of contemporary immigration control—each made up of a combination of policies, practices, and discourses—used by the majority of European states and the EU as a response to migration from the Global South to the Global North (Arnoux Bellavitis and Ripoll Servent, 2026; Boeles et al., 2025; Geddes et al., 2020; Carrera and Geddes, 2021). One arm blocks departures and pushes back arrivals, either keeping migrants away from European soil altogether or ensuring they remain in a situation of precarity once they have arrived, making them as temporary, unstable and blackmailable as possible; and the other arm removes those who have been staying in a European state for varying periods of time, with little regard for their individual conditions.

Stuck between the pincers of the war on migration and the war on welfare, non-returnable^1^ migrants are third-country nationals with an expulsion order who cannot be removed for policy reasons or due to *de jure* (legal) or *de facto* (practical) factors^2^ Absent in public debate and marginal in the development of immigration law, the issue of non-returnability affects different levels of government (national, regional, and international), a variety of subjects (governments, the civil service, state agents, migrants, civil society, international organisations), and public debate on migration. Above all, the absence of a single and shared definition of non-returnability and of clear shared norms leaves much up to the discretion of individual institutions or professionals, meaning non-returnable migrants are often subject to legal uncertainty and social instability, rights violations and unequal treatment. Across the EU countries different conditions determine the non-returnability status, developing from a mix of national policy decisions, local institutions, supranational legal sources, and factors external independent both to the authorities of the deporting state and the affected migrant. This means a non-returnable migrant's rights and living conditions change according to the state and local context in which they find themselves. Being declared non-returnable can sometimes lead to regularization, with conditions varying according to the migration status an individual is given (for example, victims of human trafficking, pregnant women, etc.). However, during their period of non-returnability, which can last for several months and sometimes some years, they are in a highly precarious, liminal situation, which gives rise to vulnerability, social risk, distress and malaise, especially if they remain in this state for long periods of time and their access to basic services is limited.

While there is a growing literature on the relationship between irregularity and forced returns (Carrera, 2016; De Genova, 2002, 2022; Strasser and Sökefeld, 2025), non-returnability needs to be studied in greater depth from a socio-ethnographic point of view focused on life conditions of non-returnable migrants. In fact, when non-returnability is addressed, it is usually through analyses of policy and legal frameworks, often comparing how different states define and manage the immigration status of non-returnability (Baldaccini, 2009; Delvino, 2020; Majcher and Strik, 2021; Carrera et al., 2025; Gines Martin, 2025). Using this approach, some authors have critically assessed policy effectiveness in relation to declared goals and variations in the implementation of returns, while also taking into account wider migration policy (Carrera and Allsopp, 2017; Czaika and De Haas, 2013). Leerkes and Van Houte (2020) and Van Houte and Leerkes (2019) revealed variation in different states' policies and practices of non-returnability, identifying four policy regimes: thick; targeted; hampered; and thin. The four regimes are characterised by a certain degree of interest of the state in enforcing returns (understood as a way for the state to obtain legitimacy in the eyes of its citizens), and capacities and resources, which are represented by both economic and human resources and bureaucratic apparatus. The first is found in the Netherlands and Norway, in which the states have a strong interest in enforcing returns and sufficient capacity and resources to do so. Under this regime, non-returnable migrants are entitled to be housed until they are returned. The targeted regime, found in Germany and Sweden, allows some groups of migrants to be exempted from enforced returns, although the states have a relatively strong enforcement capacity. The German residence permit known as a *Duldung* (a tolerated stay permit, discussed in parts 3 and 4) that is reserved for non-returnable migrants is an example of this. In the hampered regime, exemplified by Belgium and Denmark, states lack and do not allocate adequate capacities and resources for enforced returns although they have a strong interest in return enforcement. In thin regimes, as found in Italy and Spain, there is limited interest in enforcing returns, as well as a lack of capacity and resources to do so. Leerkes and Van Houte (2020, p. 333) argue that adoption of the latter regime is related to these countries' reliance on migrant labor in the underground economy, resulting in a politics of *de facto* toleration.

Despite their suspended expulsion orders, the literature has highlighted that non-returnable migrants have limited or no avenues to becoming regularized, which has a major impact on their daily lives. Living with indefinite temporal uncertainty, precarity, and suspension leaves them in a state of “limbo” (Brun and Fábos, 2015) or “stuckness” (Hage, 2009). As regards how non-returnable migrants experience the temporal dimension of living in limbo, Griffiths (2014) and Nimführ and Sesay (2019) point out that non-returnability is not simply a legal condition but also a specific temporal regime that shapes everyday life. Griffiths identifies four experiential temporalities that define non-returnability: waiting for long periods (sticky time); periods of stagnation (suspended time); periods of sudden intense activity (frenzied time); and breaks in future life plans (temporal ruptures). Similarly, in their analysis of non-returnable migrants in Malta, Nimführ, and Sesay argue that non-returnability is a condition of “permanent temporariness” produced by the interplay between enforced immobilities and migrants' attempts to appropriate mobility. Moreover, they claim that non-returnability is not simply legal and structural but “experience-based” (Nimführ and Sesay, 2019, p. 14).

Based on the Horizon Europe Project “MORE – Motivations, experiences and consequences of returns and readmissions policy (n° 101094107),” this article combines a socio-legal study and ethnographic research to analyse Italy's policy and legal framework on non-returnability and its effects on the lives of migrants. The following research questions were posed in order to analyse this phenomenon: (1) How is non-returnability defined and addressed in Italy, and how has the policy and legal framework on non-returnability evolved over time?; (2) How do non-returnable migrants experience everyday life in Italy? While the literature on the legal status of non-returnable migrants is extensive and well-established, the living conditions of these people remain understudied. Therefore, this article contributes to the existing literature by analysing the impact of migration policies on the daily lives of non-returnable migrants. Within this framework, combining a socio-legal perspective with ethnographic material, it builds on research highlighting the tension between legal provisions and everyday life, in particular shedding light on the experience of uncertainty and suspension. The article advances migration scholarship by contributing to an interdisciplinary analysis of the human and social dimensions of non-returnability.

This article is organized into four further sections. Section 2 outlines the methodology and Section 3 examines the origins, evolution, and characteristics of non-returnability within the EU and Italy, exploring the different factors that might disrupt returns and result in an irregularized migrant becoming non-returnable. Highlighting the way in which the policy and legal framework is applied in Italy, Section 4 explores the consequences its policies and laws have on migrants lives (in terms of mobility, labor, health, access to services, and so on). Section 5 discusses the broader implications of non-returnability, highlighting the need for further studies on the living experiences of non-returnable migrants.

## 2 Methodology

To understand how the category of non-returnability has been regulated over the years, we examined laws, policy documents, and reports on non-returnable migrants at the national and supranational levels issued between 1998 and 2025. In order to explore the social conditions of these migrants, within the Horizon project MORE we carried out ethnographic research between January 2024 and June 2025, including semi-structured and in-depth interviews with 9 policy experts, 19 state agents (policy implementers), 17 migrants at risk of return, and 16 agents supporting migrants at risk of being returned (NGOs, activists, etc), and two focus groups with supporting agents.

To access the interviewees, we used maximum variation sampling and snowball sampling. The first is used before data collection starts, and aims to find multiple perspectives on an issue, and ensure that those sampled come from a wide range of geographical areas. For this study, we interviewed those directly involved in implementing return policies (police officers, deportation center staff, officers in charge of refugee status determination, doctors, nurses, social workers, legal representatives, administrative staff in charge of organizing the return and in contact with migrants in detention centers, people escorting during the return as police), and those supporting migrants at risk of return (Ngos, charities, CSOs, lawyers). After data collection began, snowball sampling helped us to recruit more participants who were related to the implementation of return policies.

We developed codes through thematic analysis, which allowed us to identify and describe the relationships between themes (Boyatzis, 1998). Identified codes were grouped in two main family codes: (1) migration policies; (2) consequences on migrants' lives. The child codes of the first family were: sudden changes in Italian migration legislation; discretionality in policy implementation; administrative barriers; lack of link between non-returnability and regularization. The child codes of the second family were: precarity; uncertainty; lack of information; navigating strategies for non-returnability. One last single code was: identification of non-returnability. We adopted a “hybrid approach” (Boyatzis, 1998, p. 51) for code generation, which combines research and data-driven approaches, bringing together the researchers' thoughts, ideas and perceptions with the available data and theories already in literature. To assess the internal validity of the themes, we used constant comparative analysis (Strauss and Corbin, 1990). The data was analyzed using NVivo software, employing a conceptually clustered matrix (Miles and Huberman, 1994) to group and synthesise the data according to the themes identified, and to analyse in relation to the relevant concepts identified.

## 3 On non-returnability

Non-returnable migrants fall under article 9 of the Return Directive 2008/115/EC,^3^ which defines postponement of removal. According to this provision, EU states can postpone removal for non-refoulement related reasons or if the return decision has been suspended. Hence, non-returnable migrants are individuals whose presence is recognized by state authorities but who are not returned despite the irregularity of their legal status.

Although the EU Return Directive recognizes the possibility of postponing return, it fails to address this legal gray area in which the boundary between regular and irregular residence remains uncertain. As far as the basic conditions of subsistence are concerned, the EU Return Directive failed to set out any provisions for a protective status. The Directive requires member states to provide non-returnable migrants with a written confirmation of their situation, without specifying the type of residence status and leaving the decision to the member states' discretion.^4^ Consequently, the rights of non-returnable migrants in the EU vary from state to state. This determines a lack of clarity and homogeneity in the legal definitions of non-returnability at both national and EU level (Lutz, 2018), the absence of a shared definition of the category of “non-returnable” and the huge diversity in the situations of non-returnable migrants (European Commission, 2013^5^). Moreover, it is not always clear or predictable when a situation of non-returnability will begin or end, also due to the ever-changing and transitory nature of its underlying causes. In the “revolving door” system of contemporary migration policy (Ambrosini and Haier, 2023; Düvell, 2011; Spencer and Triandafyllidou, 2020), migrants start and leave irregular employment and gain and lose regular immigration status, as well as being subject to different laws depending on the country they find themselves in, all of which affects their legal status and social conditions. Thus, the administrative paths of migrants are not linear but often consist of repeated status transitions (Morris, 2003, 2009; McKay et al., 2011), including various attempts at regularization.

Despite these ambiguities, a non-returnable migrant can be defined as someone who has an irregular immigration status but cannot be removed, at least for the time being, either for legal, policy-related or practical reasons. Non-returnable migrants are a varied social group united by one characteristic: although they are known to national authorities, they cannot be forcibly removed from the national territory (Paasche et al., 2026). They thus have an undetermined and transitory status (Queiroz, 2018, p. 282), which can last as long as the reason impeding their return remains, which is often not foreseeable. Thus, non-returnability is, by definition, a temporary and mutable status, based on circumstances which may change over time (Farcy, 2020; Lutz, 2018). In this atypical and paradoxical situation, an individual's immigration status is simultaneously regular and irregular, making them an un(der)documented migrant.

Although the condition of non-returnability is shaped by various intersecting legal, political, administrative, and social factors (Kubal, 2013; Maes, 2011; Majcher, 2020), in this article we adopt the conventional three-part division of causes. The various possible causes that might render an irregular migrant non-returnable fall into three categories, which are not absolute and exclusive, but rather porous and overlapping: (1) those relating to policy decisions; (2) those relating to the legal or humanitarian sphere^6^; and (3) those relating to practical problems. The first category pertains to the discretionary sphere of the state and can be traced back to choices linked to constitutional provisions or the public interest rather than to supranational obligations [European Union Agency for Fundamental Rights [Fra], 2011]. For example, in Italy Article 20-*bis* of the Consolidated Immigration Act [*Testo Unico sull'Immigrazione* (TUI)^7^] grants a residence permit “for disasters” in cases of natural disasters or other particularly serious events in the country of origin.^8^

The second category of causes—those regarding elements of a legal or humanitarian nature—derives primarily from supranational legal sources, especially international human rights and EU law (Queiroz, 2018). Assessments relating to the humanitarian situation in the country of origin and the risk of direct and chain (or indirect) *refoulement*, the protection of private and family life, and the health conditions of the individual are particularly relevant (European Union Agency for Fundamental Rights [Fra], 2011). In Europe, the principle of *non-refoulement*^9^ has gradually taken on a fundamental role in the protection of the rights of third-country nationals, especially as an obstacle to forced returns (Ristik, 2017; Sevrin, 2026). A cornerstone of international refugee law, it is explicitly cited within the 2008 EU Return Directive as a fundamental consideration in assessing the legitimacy of a return (Recital 8) as well as one of the mandatory grounds for the postponement of removal (Art. 9). Under Italian law, it constitutes an absolute ban on removal in cases where there is a risk of persecution for one of the reasons aforementioned which go beyond those foreseen under the Refugee Convention (TUI, Art. 19, para 1): sex, sexual orientation, gender identity, language, individual or social conditions. Moreover, in theory it does not provide for exclusion clauses, thereby acting as a safety net regardless of the type and timing of procedure in which one finds oneself (Favilli, 2022, p. 114). A further barrier to removal in the second category is the protection of private and family life, as expressed by Art. 8 European Convention of Human Rights (ECHR) and the interpretation of the European Court of Human Rights (ECtHR). However, this right is not absolute: limitations and interferences by public authorities are possible in the interests of, amongst other things, national security, the protection of public health or morals, and the protection of the rights and freedoms of others [Art. 8(2) ECHR]. Therefore, to challenge an expulsion order on this basis, the migrant should demonstrate the existence of ties in the host country amounting to a “private and family life,” and show that these are sufficiently strong to outweigh the state's power to expel them (Majcher, 2019). Interpretative difficulties have arisen from the inconsistency of the ECtHR jurisprudence regarding the application of Art. 8 to cases of forced removal. It has produced judgments that were seemingly based more on facts than principles, developing the concept of “family life” far more than that of “private life” (Majcher, 2019).^10^ In part 4 we elaborate on the question of family life in Italy.

A further reason for the postponement of removal, still falling within the second category, is the issuing of a residence permit to victims of human trafficking and severe exploitation. In 1998, when international laws relating to human trafficking were exclusively focused on criminalizing it (Obokata, 2003), rather than creating instruments to protect victims, the Italian government introduced Article 18 of the Consolidated Immigration Act, which allowed for a residence permit^11^ to be issued to individuals who had been victims of “violence or severe exploitation.” Anchored within the paradigm of human rights and victim protection, this policy anticipated subsequent developments in international law, which, from the 2000s onwards (Gallagher, 2010, 2015), was increasingly oriented toward the protection of victims. In this case, we see the shifting of the provision of non-returnability for being a victim of trafficking to an area included between non-returnability as a result of policy, and for humanitarian and legal reasons, also pursuant to obligations arising from asylum law and the principle of non-refoulement. Furthermore, article 18-*bis* allowed for a residence permit to be issued to victims of domestic violence in situations of confirmed violence, where a “concrete and current danger” to the person's safety emerges as a consequence of their choice to escape the violence (TUI, Art. 18-*bis*, para 1, introduced by Law no. 119/2013).^12^ Recently, article 18-*ter* (inserted in the Consolidated Immigration Act in 2024) allowed for a residence permit to be issued to victims of illicit intermediation of workforce and labor exploitation.

Another reason for the postponement of removal, again within the second category, is the individual's health condition. According to the 2008 Return Directive, states should consider the health conditions of the individual concerned (Art. 5) and, after an assessment of the specific circumstances of each case, may postpone removal on various grounds including their physical and mental condition [Art. 9(2)]. This implies that, prior to proceeding with the removal of an individual suffering from a serious illness, member states “must be able to rule out any serious doubts regarding the risk that returning that third-country national results in a rapid, significant and permanent worsening of that illness or of the pain caused by that illness” (Case C-69/21, para 80). As mentioned above, under Italian law these cases fall within TUI Art. 19, which has also been subject to numerous reforms in recent years. The introduction of a residence permit for “medical treatment” limited to situations of “particular gravity” (TUI, Art. 19, para 2, lett. d-*bis*) was followed by a first extensive reform in 2020, and a further restrictive reform in 2023. In such cases, the recognition of non-returnability lasts as long as the health conditions for which the residence permit was issued. Again we see that the condition of being non-returnable is volatile and reversible.

The third category of causes—those relating to practical problems—includes all those practical impediments that render the suspension or postponement of a removal a *de facto* necessity, even where the state is under no legal obligation to suspend or postpone that removal (Queiroz, 2018). According to the 2008 Return Directive [Art. 9(2)], states may postpone removal for practical reasons that include difficulties in identifying the individual or their nationality, the absence of valid travel documents or problems obtaining them and lack of transport options. Other impediments include the absence of safe travel routes to the country of origin, the non-cooperation of public authorities of the state to which the migrant is being returned, and the non-existence of readmission agreements (European Union Agency for Fundamental Rights [Fra], 2011).

Non-returnability for practical reasons has generated atypical immigration statuses, and European countries have different approaches to these atypical immigration statuses, which condemn migrants to an indefinite legal limbo. In some cases, a temporary residence permit is granted, occasionally linked to residency requirements; in other cases, no particular status or certificate attesting to the suspension of removal is issued, rendering individuals in this situation indistinguishable from irregular migrants who are not known to authorities. In other cases “tolerated” statuses are permitted, such as the *Duldung* in Germany (under the 1965 Immigration Act), the *Začasno ZadrŽevanje* (temporary suspension of removal) in Slovenia (Aliens Act 2011) and “special immigration status” in the UK (under the 2007 UK Borders Act).

In Italy, those who cannot be returned for practical reasons do not receive a specific immigration status. If removal is not feasible and there are not “reasonable prospects” (TUI, Art. 14) that it may happen, authorities order the migrant to leave Italy independently through their own means within 7 days. This creates a situation of *de facto* non-returnability that is not certified by the authorities. Thus, despite having ample resources, the state places the responsibility for return on the shoulders of the migrants themselves, requesting that they go back to their countries of origin using their own limited means. The state assumes that the migrant will comply with an order which is not only difficult to put into practice, but which tends to run counter to their own desire not to return to their country of origin and to instead stay in Italy.

### 3.1 A volatile and debated provision: the special protection in Italy

The residence permit for special protection is unique to Italy, and falls within the group of causes relating to the legal or humanitarian sphere, although it significantly overlaps with the category of causes relating to policy. The residence permit for special protection, established in 2018 with Law 132/2018 (duration 1 year), comes from the system of humanitarian protection, which was introduced in 1998^13^ (and remained in force until 2018) to provide protection to those who faced serious humanitarian issues but did not qualify for asylum or (from 2008) subsidiary protection. In the 2015–2018 period, humanitarian protection became fundamental for guaranteeing a form of protection to migrants in vulnerable situations, who had been traumatized along migration routes or who were victims of human trafficking or exploitation but were not yet formally recognized as such, and was also used to safeguard personal and family ties (Guidi et al., 2023).

Instrumentalized by far-right parties who described it as an “Italian anomaly” that “gave residence permits and reception to illegal migrants,” the institute of humanitarian protection was abolished by the Italian government in 2018.^14^ This removed safeguards for all those who would have previously been granted such a permit, including asylum seekers who had reached a “certain level” (not precisely defined) of social integration based on the extensive interpretation of the law (Ferri, 2021). Alongside the abolition of humanitarian protection, provisions were introduced in the same year prohibiting the return of those in “vulnerable categories,” defining different types of temporary protection. Examples included “medical care,” “natural calamities,” “special cases.” The scope of humanitarian protection was thus significantly reduced to a few categories that did not fully represent the variety of individuals who found themselves in a situation of vulnerability without being formally recognized as such.

In 2020,^15^ the Italian government in practice reintroduced humanitarian protection. The residence permit for special protection was modified to allow for a residence permit (duration 2 years) based on private and family life.^16^ For the first time in Italy, a migrant's level of social inclusion was used as an assessment criterion when deciding whether to grant a residence permit. This formally introduced into law what had been anticipated in jurisprudence relating to humanitarian protection (Ferri, 2021). It has often been defined as protection “by integration,” since it required that the applicant had proof of social integration, including a history of living in Italy, stable employment, a home, a family, had attended Italian language classes or had past experiences of volunteering.

By leveraging social and labor inclusion in Italy, residence permits for special protection allowed irregular migrants to be considered non-returnable and thus to acquire a regular immigration status. This was the first provision of its kind in Italy, which, unlike other European countries, had never before provided for regularization based on these criteria. However, the description of special protection in law was intentionally left broad and thus susceptible to differing interpretations by the local commissions responsible for the recognition of international protection. At the same time, although case law has offered guidance in defining and contextualizing the letter of the law, this has occasionally been contradictory,^17^ resulting in a high level of discretion in administrative practices and differences in application by the aforementioned local commissions and local police departments (*questura*).

In 2023, the Italian government restricted the residence permit for special protection.^18^ The reformed special protection became much more limited in duration (again 1 year) and in scope, repealing protection “by integration,” with a consequent narrowing of safeguards for migrants, who were thus at greater risk of irregularization (Zorzella, 2023). All international protection applications were evaluated without taking into account the individual's life in Italy, thus impacting on their right to a private and family life. The residence permit for special protection can only be renewed if the conditions under which it was granted still apply and cannot be transformed into a work permit, although it does grant the holder a right to work in the country. In November 2025 the situation changed again, when Italy's highest appeal court (the *Corte di Cassazione*) made applicable special protection based on the right to a private life, deciding that international and constitutional obligations had to be observed. And so, with the reintroduction of this provision the scope of regularisation expanded again. As we will see, this “accordion” type situation of alternating compressions and expansions of the law means that its impact on the lives of migrants can vary hugely depending on the period in which they apply for regularisation.

As we have seen, the Italian policy framework governing the condition of non-returnable migrants is heterogenous and mobile. Non-returnability is subject to tensions and changes over time following legislative amendments that alternatively broaden and limit it. The circumstances shift over time within a complex legal framework, with huge consequences on migrants' daily lives. This framework is interpreted and applied on a daily basis by different public sectors (healthcare, employment, home affairs, education, and so on) which, due to its ambiguities, end up using their discretion, leading to arbitrariness in their decisions.

As we have seen, the condition of non-returnability is not adequately defined and regulated, since there is no shared definition of who a non-returnable migrant is. Furthermore, non-returnability has a contradictory temporality: it is temporary yet indefinite; it might lead to a residence permit or might condemn the individual to a never-ending limbo between an irregular and regular status. This has a major impact on migrants' employment and living conditions, on their access to services and on their social relations. In short it forces people into a limbo between regularity and irregularity, staying and being returned, greatly affecting all aspects of their lives.

## 4 Living in limbo

The 2008 Return Directive indicates the need to address the situation of non-returnable migrants, but leaves it to the discretion of individual member states to identify the basic conditions for their subsistence (Recital 12). It does however guarantee certain safeguards pending return in relation to family unity, the right to health, the right to education for minors, and the special needs of vulnerable individuals (Art. 14).

In 2014, the European Commission pointed out that non-returnable migrants found themselves in a condition of administrative limbo, often forced to rely on services offered by civil society organizations to meet their daily needs (European Commission, 2014, 2017). Subsequent policy provisions, including the new proposal for an EU Return Regulation presented by the European Commission in March 2025, failed to further define, clarify and explain the rights of non-returnable migrants. They instead repeatedly stressed the complete discretion of member states concerning the “written confirmation” issued to non-returnable migrants to certify that they are indeed non-returnable, as well as there being no obligation for authorities to issue a residence permit in such cases. These provisions also make no further comment on the right to work, housing, or adult education. The failure to regulate these aspects has multiple negative consequences on the lives of non-returnable migrants, excluding them from the labor market—pushing them into the underground economy where they are at risk of labor exploitation,—and forcing them into precarious living conditions, health poverty, welfare dependence on NGOs and informal aid, and a condition of liminality and suspension (Carrera and Stefan, 2020; Schoukens and Buttiens, 2017; Rosenberger, 2019; Delvino, 2020).

In Italy, the state's decisions on the migration status of non-returnable migrants have consistently been marked by inaction and negligence. For instance, no residence permit was created to provide for the temporary regularization of non-returnable migrants. At the same time, as discussed above, non-returnability as a result of policy or due to humanitarian concerns have changed over the years. Our empirical research has highlighted that the rights granted to those officially recognized as “non-returnable” have been extended and compressed like an accordion depending on the political coalitions governing at any given time or on the discretion of local police departments. Indeed, as one judge stated:

“That safety net of special protection, humanitarian protection, and special cases. [These provisions] were amended in quick succession: in 2018, [the net] was tightened; in 2020, it was reformed by the center-left government; in 2022, it was tightened by another law… the legislation has been amended so rapidly, changing the requirements and also the possibility of renewing residence permits, of stabilizing those who had special protection… The latest legislative changes in 2023 modified the requirements and again called into question all those who had achieved a certain status, a certain level of integration, social inclusion and employment. And many have been thrown out [of the system] again. We have cases of people who are on their third appeal.”^19^

In Italy, whether or not they are issued with a residence permit has a fundamental effect on the living conditions of non-returnable migrants, as the enjoyment of rights and access to services are dependent on it. Unlike other countries such as Germany, the UK, and Slovenia mentioned above, in Italy there is no “tolerated” residence permit specific to cases of non-returnability. Where non-returnable migrants are not issued with a residence permit, this leads to their working in the underground economy and weak social inclusion, characterized by precarity and insecurity. To obtain a residence permit, they must fall precisely within the types provided for by the law.

In addition, Italian migration policies have been largely developed and implemented through administrative circulars, which are issued by government departments to the central and local civil service. These are not necessarily public and are often difficult to access. They are not sources of law but secondary norms that do not provide a general political direction and leave ample margins for interpretation in their application, paving the way for administrative arbitrariness. In this context of the “administrativisation of migration policy” (Gjergji, 2013) and “formal informality” (Cardwell and Dickson, 2023), non-returnable migrants find it hard to know their rights and decisions about these rights are often at the discretion of public officials and policy implementers. As a supporting agent told us:

Sometimes when a person is unreturnable they go to the police station and the police station doesn't assess the grounds for their being unreturnable. That is, if it favors rejection, it rejects their application. In this case, accompanying the person to the police station can be helpful, because it is also useful to try to invoke the previous legal grounds for unreturnability. For example, there was a case in which a person who had a residence permit for medical treatment asked for it to be converted into a residence permit, but the state refused the request because there had not been a final judgment, the trial was still in progress. After the refusal they didn't want to give them a new appointment at the police station to apply for the residence permit for medical reasons, they wanted to return them immediately. And so we went to the police station, showed them all the documentation and said, “look, you're making a mistake.”^20^

As our research shows, the rigid categories which migrants have to fit into in order to be formally identified as “non-returnable” have important repercussions on their daily lives. Non-returnability is not synonymous with regularization, not even where it is based on humanitarian concerns or for reasons of policy, due to the great number of situations in which the conditions for obtaining a residence permit cannot be met. Degani et al. (2023) point out that a pregnant woman with no passport could encounter difficulties in obtaining a residence permit for medical reasons, even if they have a legal right to it, because a passport is a necessary prerequisite for obtaining it. In our research, we encountered the case of a minor who was exploited in underground economies: albeit formally recognized as non-returnable, these persons could not obtain a residence permit unless they pressed charges against the exploiters or engaged with social services.

The fieldwork reveals that the presence of family ties is a frequent cause of non-returnability. However this does not always come with a right to a formal certification and the consequent issuing of a residence permit, as a state agent told us:

“Often even the necessary conditions, such as suitable accommodation, preclude the possibility of obtaining this type of residence permit, or proving genuine ongoing cohabitation with people who often work in areas that are outside of the city and so are not present when there are police checks.”^21^

Confirming this, and in line with the legislation,^22^ interviewees stressed that the suspension of a removal order by the judicial authority does not automatically provide for regularization, unless the person submits an application for a residence permit. Otherwise, the person will only be certified as non-returnable until the date of the court hearing.

Precarity and uncertainty are evident in cases of non-returnability for practical reasons. As a policy expert observed, “the state shows its incompetence: in 18 months of detention it has not been able to remove a person, despite having means, political and diplomatic power, and resources, and it expects the foreign national to compensate for its failure in seven days.”^23^ As we have said above, this difficulty is compounded by the fact that, in the 7 days during which they should hypothetically be organizing their return, migrants are not issued with a temporary permit of any kind.

Knowledge of potential ways to get out of irregularity and avoid deportation is essential: applying for asylum or for a residence permit requires that the migrant is aware of their options. In this context, our research confirms the importance of intermediaries such as social caseworkers, who are fundamental for navigating the knotty intricacies of immigration law and government bureaucracy (Bonizzoni et al., 2025). However, the need to find information may lead them to rely on informal or unauthorized intermediaries (organized crime, fixers, compatriots, or lawyers) who can profit from and take advantage of migrants' needs, sometimes in veritable scams. These subjects “fill” a void created by the negligence of state institutions, as a policy expert told us:

“In the activities we carried out with irregular migrants we constantly encountered a lack of information. Information doesn't reach people, they don't know where to get information, they are scared, and they no longer trust institutions. […] Many irregular migrants could be regularized, but they don't know that.”^24^

Intermediaries are essential not only for accessing regularization procedures, but also healthcare services, housing, employment and educational services. The anomalous status of “non-returnable” often means they avoid institutional settings for fear of being identified and deported. This means they miss out on services and do not go to public spaces, relegating themselves to the margins of society to avoid being seen.

### 4.1 Seeking invisibility, visualizing non-returnability

Our research shows that the search for invisibility and strategies of invisibilization are survival mechanisms adopted by non-returnable migrants. These include avoiding hospitals, employment agencies, job centers, language schools, and estate agents in order to minimize interactions with state institutions. A supporting agent shared the story of a Tunisian unaccompanied minor, referred from the anti-trafficking hotline for suspected involvement in an exploitation ring and, for this reason, potentially non-returnable for reasons of policy or due to humanitarian concerns:

When we reached this young man we discovered that this was the last of his problems: he had been completely isolated for at least a year, trapped, worrying that he would have problems if he was noticed. He had built a makeshift shack in the countryside surrounding [the city] and lived there, working when he could find daywork… […] He kept saying: “I'm scared, what if I go out and I meet the police, or if I get a train and they stop me?.”^25^

This self-invisibilization prevents non-returnable migrants from accessing their rights and gaining protection, forcing them to rely on informal networks. A supporting agent^26^ working in a low-threshold healthcare service for those without a health card, stated that, due to recent changes in legislation and the resulting restriction of safeguards, many migrants are afraid of accessing their services, even if they have been in Italy for many years and are in a situation of non-returnability. This is because they are afraid of being intercepted and receiving an expulsion order or, worse, of being immediately detained in a deportation center.

These processes of concealment, self-invisibilization and self-marginalization combine with difficulties in communication and understanding between public services and non-returnable migrants. As a supporting agent observed:

“There is always a need for specific information, for a relationship to be built with the person, who will trust us if we give them the message: “Even if you don't have a residence permit at the moment, but you need to visit us, don't worry, you can.” This information is not widely shared, also because the majority of migrants we work with don't know the context, they don't come from a context in which a widespread healthcare system exists. So even understanding our society is difficult.”^27^

Aside from the need for extensive information on rights and services, interviewees also highlighted issues with the recognition of non-returnable migrants by state institutions and their identification by professionals who are not adequately trained. Like migrants at risk of return and irregular migrants, non-returnable migrants frequently encounter institutional invisibility due to their exclusion from official registries and social services, as also noted by Özdemir (2024). And while factors that could result in non-returnability are sometimes evident (for instance, pregnant women), sometimes they could be difficult to identify, such as when they are the cause of emigration or are connected to involvement in trafficking and exploitation. In these cases, social caseworkers play a significant role. However, this requires adequate skills, as a supporting agent in an anti-trafficking body told us:

“We mainly see Bangladeshis […] and almost all of them would have been trafficked. It is a serious problem that they are not then flagged to the commissions to be granted international protection. And their claim is also refused as manifestly unfounded with an expulsion order attached, and this has become a huge problem. Because notice for expulsion is now given at the same time as the application for international protection is rejected, so it is difficult to offer them protection.” (see text footnote 25)

The situation inside deportation centers further exacerbates these difficulties. The deficiencies and structural violations of these centers (Esposito et al., 2022; Manghi, 2021; Cocco et al., 2024) add to the lack of information, problems in accessing legal support, and the limited ability of professionals to identify non-returnable migrants. It is fairly unlikely that a migrant held in a deportation center would apply for asylum, as they have limited access to legal information, inadequate assistance and face various administrative obstacles (ASGI, 2023). A judge told us:

“Justices of the Peace^28^ have a bit of a training problem. [...] The laws have to be studied. And it shouldn't be that if the defense tells you something, you say: “Alright, that's what the defense says.” [but you do not consider it]. No – you check whether the legal rule is correct and you examine the underlying situation. The problems in the deportation centers [...] also include the lack of preparedness of the defense. Frequently, asylum seekers are assisted by a court-appointed lawyer who often does not know the subject matter. However, when the lawyers do know the field, they raise solid objections that quite rightly force us to study the issues brought before us.”^29^

## 5 Final thoughts on suspended life

Thus, in the hierarchy of categories into which migrants are divided by national and international legal frameworks (Morris, 2002; Lockwood, 1996), non-returnable migrants are just one rung above irregular migrants—the undocumented *tout court*.

Our research has shown that “non-returnable” status is heterogeneous, unstable and changeable. People in this category are constantly subject to administrative changes and variations in their social status due to modifications in national legislation and in policy implementation at a local level, as well as changes in the labor market and the housing situation. We have seen that the legal limbo in which people in this category find themselves often translates into a veritable social limbo, in which their social life seems to be put on hold. The word limbo comes from the Latin word *limbus*, meaning edge, border, hem or fringe. In medieval theology, limbo is the border zone between hell and paradise, serving as a metaphor for a margin or a situation of suspension, waiting or uncertainty. Our study has highlighted three factors: the indeterminacy and reversibility of this legal status; its temporality; and the invisibility it creates.

As regards the first aspect, the rights and living conditions of non-returnable migrants vary according to the historical phase and the country and local context in which they find themselves. This may mean an individual is granted a residence permit, while at the same time being condemned to remain in legal limbo, leaving them at the margins between a regular and an irregular status. Furthermore, the beginning or end of a situation of non-returnability is not always foreseeable or certain, partly due to the transitory nature of its underlying causes. This instability results in atypical migration statuses, which is reflected in the term “tolerated statuses” used by various states to describe the condition of non-returnability. In this anomalous and paradoxical situation, regularity and irregularity coexist within the individual's migration status, with non-returnability persisting for as long as the conditions justifying the residence permit remain in place.

In Italy, the condition of non-returnability is subject to constant tensions and changes due to legislative amendments that alternately extend or limit it. It is subject to different interpretations and applications by the various public sector institutions (in health, social welfare, home affairs, education, etc.), which apply it with significant discretion and arbitrariness. Thus, non-returnability in Italy suffers from variability and vagueness both in its legal definition and in its application. We have also pointed out that the rights of non-returnable migrants are expanded and compressed like an accordion, depending on which government is in power or on decisions made by the local police department.

All of this has multiple consequences for the daily lives of non-returnable migrants, including on temporality. Although non-returnability is temporary, the duration of non-returnability is, by definition, not given, for it is always unclear when a situation of non-returnability will begin or end. It lasts as long as the obstacle impeding return, which is often unpredictable: the transitory nature of non-returnability is caused by factors that are themselves transitory. Obviously, this has a direct and devastating impact on the non-returnable migrant, who, finding themselves in a temporal limbo struggles to make life plans.

The third and final aspect is the invisibility arising from the state of suspension and uncertainty that non-returnable migrants experience on a daily basis due to the social, existential, and psychological limbo they are caught in.

Authorized to remain in a country for a limited period that could be significantly extended, often lacking a residence permit due to their non-returnable status and unable to apply for one because they do not meet the requirements set out in immigration legislation, non-returnable migrants are a veritable living paradox. This liminal situation is a source of vulnerability and social risk, as well as distress and unease, especially if it is long-lasting and if access to services is limited. It can drive non-returnable migrants into the informal economy, offers them only limited opportunities for social inclusion and is characterized by a lack of access to healthcare, dependence on NGOs, and invisibility.

Although this invisibility is ultimately the result of laws which deny them access to certain services and state institutions that refuse to grant them a recognized legal status, people in this condition often also try to make themselves invisible. Our research has shown that non-returnable migrants often adopt strategies to render themselves invisible as a survival mechanism to minimize interactions with state institutions, particularly with public officials and the police. However, these strategies of avoidance, self-invisibilization and self-marginalization hinder them from accessing services, safeguards and their own rights, forcing them to resort to informal networks and, at times, illegal channels.

Labeled by the state as tolerated irregular migrants and caught in a process of continuous and alternating inclusion and exclusion, non-returnable migrants are trapped in a paper swamp, where they are dragged down and their social vulnerability is increased.

## Author's note

This article is the result of a collective, joint, and indivisible effort. However, should individual attributions be assigned: Francesca Cimino wrote sections 2 and 4; Beatrice Grasso wrote section 3; Fabio Perocco wrote sections 3 and 5. Section 1 was written jointly by Francesca Cimino and Fabio Perocco.

## Data Availability

The datasets presented in this article are not readily available because they include interview transcripts. Requests to access the datasets should be directed to the corresponding author.

## Footnotes

1 Although the term “non-removable” prevails in the existing literature, in this article we use the term “non-returnable” because it is less dehumanising.

2 For statistics on orders of expulsion and forced expulsion, see Eurostat https://ec.europa.eu/eurostat/statistics-explained/index.php?title=Enforcement_of_immigration_legislation_statistics#Non-EU_citizens_ordered_to_leave_the_EU.

3 The Directive is being recast as a Regulation. However, at the time of writing, the final legally revised text had not yet been officially published.

4 Directive 2008/115/EC of the European Parliament and of the Council of 16 December 2008 on Common Standards and Procedures in Member States for Returning Illegally Staying Third-Country Nationals, Recital 12.

5 The European Commission has published no further reports on the implementation of the 2008 EU Return Directive since 2013, despite previously planning to do so.

6 The first two categories are sometimes conflated in the literature, given the high degree of potential overlap between policy considerations and those based on humanitarian and compassionate grounds [Platform for International Cooperation on Undocumented Migrants [Picum], 2022].

7 Legislative Decree 286/1998.

8 The introduction of a residence permit “for disasters” following the abrogation of humanitarian protection (repealed by Law Decree 113/2018) aimed to cover those situations of “contingent and exceptional calamity” which do not allow for a safe return and stay in the country of origin (TUI, Art. 20-*bis*). The uncertainty generated by the frequent reforms to humanitarian protection has not spared this safeguard, which had been reformed expansively in 2020 (Law Decree 130/2020), allowing for a wider interpretation (Rossi, 2021), and subsequently restricted again in 2023 (Law Decree 20/2023) through a reform which not only reduced the margin of interpretation, but also eliminated the possibility of converting this permit into one for employment purposes (TUI, Art. 20-*bis*, para 2), thus preventing beneficiaries of this provision from prolonging their stay on the basis of circumstances unrelated to the situation in their country of origin.

9 The *non-refoulement* principle is enshrined in Article 33 of the 1951 Convention, which is also binding on states that are party to the 1967 Protocol. Article 33(1) of the 1951 Convention states: “No Contracting State shall expel or return (‘refouler') a refugee in any manner whatsoever to the frontiers of territories where his [or her] life or freedom would be threatened on account of his [or her] race, religion, nationality, membership of a particular social group or political opinion.” In the Council of Europe's ‘European Convention for the Protection of Human Rights and Fundamental Freedoms' of 1950, the principle is expressed through Article 3 (“Prohibition of torture”), 2 (“Right to life”), and 4 of the protocol 4 (“Prohibitions of collective expulsion of aliens”).

10 A further difficulty arises from the tension between safeguarding individual rights and protecting public order, especially when this relates to cases concerning foreigners with criminal convictions [European Union Agency for Fundamental Rights [Fra], 2011].

11 Especially in the early 2000s, human trafficking and severe exploitation did not only affect migrants. This provision was created to ensure the protection of all victims, without discrimination.

12 The practical implementation of this norm has been the object of criticism by, among others, the Group of Experts on Action against Violence against Women and Domestic Violence, who argue that being bound by the existence of a serious and present danger to the victim's safety, the safeguard applies solely to high-risk situations, excluding all other manifestations of violence (Garisto, 2015). Moreover, the requirement that the danger be current and severe connects the risk exclusively to physical violence, thereby excluding all those forms of psychological or economic violence for which these two prerequisites would be difficult to prove, despite them falling within the definition of intrafamily violence (Biaggioni and Pirrone, 2018, p. 67).

13 TUI 1998, Art. 5, para 6.

14 Law Decree 113/2018.

15 Law Decree 130/2020.

16 Art. 8 ECHR: the right to respect for private and family life, home and correspondence.

17 In Judgment no. 24413 of 2021, the Joint Chambers of the Italian Court of Cassation asked the judge to perform a comparative assessment between the level of integration achieved in the host country and the situation in which the individual would find themselves if they were to be returned to their country of origin. Conversely, certain judgments delivered by the European Court of Human Rights (ECtHR) on the basis of Art. 8 ECHR stipulated that seven years was the minimum period that a person had to live in a territory to be considered to be integrated, although this was not the sole criterion.

18 Law 50/2023.

19 IT-IA-03.

20 IT-FG-SA-01.

21 IT-IV-SA-01.

22 TUI, Art. 13, para 8.

23 IT-IV-PE-04.

24 IT-IV-PE-08.

25 IT-IV-SA-02.

26 IT-IV-FG-02.

27 IT-IV-SA-07.

28 Justices of the Peace are non-professional magistrates. They are the judicial authority responsible for validating and extending detention in deportation centers and adjudicating appeals against administrative expulsion orders, thereby exercising judicial oversight over restrictions on migrants' personal liberty.

29 IT-IV-SA-03.
